# Corporate social responsibility and firm performance: Case of Kazakhstan

**DOI:** 10.1016/j.heliyon.2024.e31580

**Published:** 2024-05-21

**Authors:** Maya Katenova, Hassan Qudrat-Ullah

**Affiliations:** a KIMEP University, Bang College of Business, Almaty, Kazakhstan; b York University, Keele Campus, Toronto, Canada

**Keywords:** Corporate social responsibility, Profitability, Financial performance, Kazakhstan, Emerging economy

## Abstract

The research seeks to find a relationship between Corporate Social Responsibility (CSR) and companies' performance. Studied variables were measured and analysed using a sample of companies listed on the Kazakhstan Stock Exchange (KASE). The study employed the regression model and least squares technique as the primary analytical tools. CSR is examined in conjunction with variables such as Return on Assets, Return on Equity, Market Value, and Net Profit Margin. As a result of the research, it was found that firm performance and CSR relate to each other in the studied companies. The research found a positive correlation between CSR practices and Net Profit Margin in Kazakh companies. While this study focused on a single country, its methodology can be applied to research in other emerging and developing nations. The primary contribution of this research is the examination of the relationship between firm performance and CSR practices in the post-Soviet emerging market of Kazakhstan.

## 1 Introduction

Economically, developing a Corporate Social Responsibility (CSR) strategy is integral to a company's successful functioning as it strengthens societal ties. It stands as a crucial driver of economic growth for both individual businesses and nations at large. In today's global business landscape, including that of the Republic of Kazakhstan, it's essential to consider the key stakeholders. Consequently, the adoption of CSR practices fosters a connection between the company, its stakeholders, and improved operational efficiency.

The concept of "corporate social responsibility" (CSR) has evolved to represent a business management model based on the inherent duty of companies, stemming from their societal function, to implement policies and practices that contribute to sustainable societal progress [1]. CSR includes a broad array of social innovations, covering everything from environmental stewardship to advancing the social interests of diverse population segments. For example, research [2] highlights the role of CSR in fostering the social development of particular countries. Another group of researchers [3] argues that CSR primarily serves as a social goal with specific competencies for societal self-organization. Such a conclusion was made from the analysis of a survey of managers – 324 CEOs from developing countries.

It's crucial to recognize that organizational ethics hold a significant place as both a moral obligation and a social responsibility toward citizens and society [4]. It is well-established that supporting education, science, and ensuring a stable standard of living for all citizens are essential for a country's economic development [5]. This is precisely what CSR research in developing countries is paying attention to. The Republic of Kazakhstan is not an exception [6].

Firms in emerging economies tend to prioritize CSR activities, as they are essential for enhancing their image and attracting foreign investment. Companies in emerging markets promote themselves to attract more foreign investment and raise capital. It is universally acknowledged that investors favor companies with strong CSR practices. We contend that companies engaged in more CSR practices exhibit superior capabilities compared to those with fewer initiatives. There is a robust connection between financial performance and CSR activities. Furthermore, it should be emphasized that the connection between financial performance and CSR activities is more pronounced in countries that are on the path of economic development compared to countries with well-developed and stable economies [7].

Applying the institutional theory of CSR, researchers [8] conducted surveys among representatives of 800 firms. These surveys revealed that individual households are more actively involved in CSR practices compared to corporations that give less attention to this aspect.

Kazakh companies have some of the highest ESG (environmental, social, and governance) ratings in Central Asia. For example, KazMunaiGas Company achieved an ESG score of 28.4, placing it among the top 270 companies globally with the highest ESG ratings, as reported by Sustain Analytics Agency in December 2022.

Recently, India and the People's Republic of China have conducted the most research in the field of CSR application – the most active countries in the world regarding development rates. Unfortunately, other countries in Asia (South and South-East regions) do not give CSR due attention [9]. Hah and Freeman [6] note that while studies of CSR are of interest to countries where this indicator aligns with their policy context, they are not always integrated with financial analysis and accounting metrics. Typically, the focus is on profitability, drawing parallels with CSR, even though these two aspects are interdependent and can significantly influence each other. After all, it is profitable for companies that can allow CSR because it is a high-value activity, and vice versa - CSR will increase the company's profitability. This was designated by Miethlich and Šlahor [10]: CSR is effective on a large scale in large multinational companies and corporations. CSR impacts business, improves profits and leads to new developments in the company. Similar trends are beginning to manifest in the Republic of Kazakhstan.

The Republic of Kazakhstan is a rapidly developing post-Soviet country and serves as an example of an emerging economy Since gaining independence in 1991, Kazakhstan has faced significant challenges such as hyperinflation, currency devaluation, and banking crisis. However, the Republic of Kazakhstan also experienced economic growth in early 2000. It happened during the accelerating oil price, which lasted from early 2000 until the end of 2008. In this context, numerous successful oil and gas corporations operate in the Republic of Kazakhstan. Yet, there has been no research assessing how profitability impacts CSR within this region. This groundbreaking study explores the relationship between CSR and company profitability in emerging markets.

Orazayeva and Arslan [11] reviewed possible obstacles to CSR analysis and identified key limitations, including weak stakeholder activity, corruption schemes, lack of proper state control, etc.

Conversely, existing research indicates that religious traditions, historical context, globalization, and governmental institutional gaps are the primary factors influencing CSR studies. When this framework is applied to the Republic of Kazakhstan, it is evident that these drivers and constraints are equally applicable within the country.

The points highlight the relevance of the topic, especially from a developing country's study perspective. The combination of implementing CSR practices and simultaneous measures for economic development and stabilization processes remains a relatively understudied area due to the limited international visibility of such countries.

Despite growing awareness of Corporate Social Responsibility and its potential effects on business performance, there is still a notable lack of understanding about the precise relationship between CSR and profitability, especially in emerging markets like the Republic of Kazakhstan. The scarcity of empirical studies on how CSR activities influence the profitability of companies in Kazakhstan is significant, considering the country's distinct economic challenges and periods of notable growth. This study seeks to fill this gap by shedding light on the specific interactions between CSR and profitability within Kazakhstan's unique economic environment, potentially guiding CSR strategies and economic development policies in similar emerging economies globally.

## 2 Literature review

Corporate social responsibility initiatives and their impact on corporate financial performance have been extensively studied by scholars across the globe. The term encompasses the ways in which a company contributes to environmental preservation, such as reducing air pollution and issuing green bonds. Concurrently, environmental, social, and governance (ESG) initiatives address social aspects, including employee care through the provision of favorable working conditions, effective human capital management, and robust health and safety programs.

### 2.1 ESG initiatives and financial performance

The researchers in the study [12] demonstrated a sustainable relationship between ESG activities and the financial performance of enterprises. Their analysis was based on a two-stage model applied to 403 U S. companies over the period from 2006 to 2011. Features of the ESG impact activities emphasising the enterprise's market value help companies increase their rating in the market. Another result was also derived, which allows presenting the transparency of ESG activities as an effort to justify unnecessary excessive investments in one's activities. The paper [13] found confirmation of the dependence on ESG investing and increasing ESG market value. For this purpose, 4886 companies from developing and some developed countries were studied. A two-stage least squares model was used for the proof, and the factors affecting the specified dependence were identified. For example, investing in environmental protection helps attract investors because a joint effort reduces dangerous emissions. Moreover, financing social initiatives increases labour productivity and the company's value. Here, the «equals » sign is put between labour productivity and a favorable climate in the team.

However, the results are not entirely clear-cut in the analysis mentioned. For instance, Duque-Grisales and Aguilera-Caracuel [14], in their examination of the financial statements of 104 multinational companies headquartered in Latin America from 2011 to 2015, identified a negative relationship between ESG activities and financial outcomes.

Although the authors point out that such a trend may be a feature of Latin American countries. This is due to the influence of their socio-cultural and ethnic practices. ESG activities are not always prioritised because, with limited financial resources, the emphasis shifts to needs more evident in a developing society. Yet, in the case of investment and improvement of financial activity, ESG projects begin to increase their positive impact. Duque-Grisales and Aguilera-Caracuel [14] emphasized in their paper the fact that foreign investors can finance ESG as a separate item.

ESG researchers also did not ignore the sustainable development of companies and the economy as a whole. Notably, this is noted in the work of El Alfy and Weber [15], who identified the ESG complex as a lever for corporate reputation and economic, social, and environmental results while analysing CSR as a phenomenon.

### 2.2 CSR and financial performance

Previous empirical research on CSR and profitability has yielded mixed results. Studies [[16], [17], [18]] have demonstrated a negative relationship between CSR and profitability. In contrast, research [[19], [20], [21]] supports the existence of stable causal relationships between CSR and firm performance, indicating a positive correlation. Further, studies [22,23] have found a strong positive relationship between CSR and profitability.

The work [24] also proved a positive relationship between CSR and the company's profitability. Moreover, the emphasis is on corporate social responsibility – the higher it is, the higher the profitability. This is also confirmed in work [25]; the proof is made using the enterprise's credit rating. Although the researchers Margolis and Walsh [26] showed in their work that this is a controversial issue because about half of the companies do not demonstrate a correlation between CSR and profitability.

Researchers Mochales and Blanch [27] proved the dependence on forming the company's brand capital precisely through CSR initiatives and note that all this, in the aggregate, affects business efficiency. The research [28] corroborated that CSR and financial results have a sustainable systemic relationship. This conclusion was obtained based on the results of 191 enterprises analysis from South Korea through the profitability and value of companies according to the index of the Korea Economic Justice Institute for 2015. The strong correlation made it possible to state that the growth of assets and CSR allows for a positive assessment of the company's activities through the ESG triad.

Waddock and Graves [29] also emphasise the indicators of past periods when analysing CSR. But researchers [30] refute the statement [29] and suggest considering CSR as a particular type of activity because ESG should not affect financial results in any way.

Currently, financial performance and CSR activities are garnering significant attention from scholars. Using traditional statistical methods, a relationship between these two variables is identified. However, employing a time series fixed effect approach reveals a weaker correlation between CSR and financial performance.

Meanwhile, researchers [31,32] conducted a meta-analysis to explore the relationship between corporate social performance and both accounting-based and market-based performance indicators. Their findings indicate a strong correlation between corporate social performance and accounting-based indicators, whereas no significant correlation was found with market-based indicators. Further research focusing on the link between corporate social responsibility and accounting-based performance measures has consistently shown positive results [17,18,33].

Among the interesting CSR studies, one's can mention the work of Сochran and Wood [34], who analysed corporate social responsibility depending on the level of asset control by age. Mainly, it was determined that the management of companies operating for a long time on the market pay less attention to CSR than modern, recently established enterprises. Researchers [35] examineed Swiss corporations operating in domestic and global markets and noted that smaller enterprises do not emphasise CSR practices. Also the same is confirmed in work [36]. Still, the attention to CSR is explained by the attitude of enterprises to some organisational parameters, in particular, internal and external communications. It is worth noting that the mentioned features are typical for the Republic of Kazakhstan.

Orlitzky [37] underlines that company managers began to understand the importance of social and environmental actions for the systemic perception of the organisation in the market. Nonetheless, some organisations do not share this point of view because CSR projects lead to additional expenses. Therefore, the question remains to be debated.

### 2.3 CSR studies beyond borders

The CSR phenomenon has been studied in the following scientific directions.

• Labour ethics and CSR [38]. The obtained results are ambiguous, but identifying the significance of activities through the prism of CSR has a positive effect on labour ethics.
• CSR initiatives and employee commitment [39].
• CSR motivation and compliance with ESG goals and objectives with an impact on reputation and behaviour [40].
• The connection between HR and CSR [41].
• CSR in the corporate strategy [42].
• CSR and executive rewards for efficiency implementation [43].
• Strategic management and correlation with CSR [44].

It is worth noting that CSR studies have gone beyond the boundaries of one company or country. In the process of work, the following was investigated.

• The Latin American companies' reputation through the prism of CSR [45].
• CSR impact on investment activity in Western Europe [46].
• CSR features in Scandinavian countries [47].
• Peculiarities of implementing CSR practices in the Philippines [48].
• CSR reporting in Arab countries [49].
• BRIC countries and CSR initiatives [50].
• CSR in some multinational subsidiaries in the Federal Democratic Republic of Ethiopia [51].
• Foreign CSR practices for a Japanese multinational company [52].
• CSR activities in India [53].

In analysing CSR practices in Kazakhstan, it should be noted that the national model of CSR is not yet sufficiently developed. Altaibayeva, Khamzina, and Baltabayeva note that the Kazakh model of CSR is characterised by active state participation in the implementation of several initiatives (socio-economic and environmental). In addition, the Republic of Kazakhstan has a strong state influence on regulating the corporations' activities, appropriate incentives, benefits, and levers, including implementing CSR projects [54].

### 2.4 Hypothesis development and study objectives

Despite the extensive literature on the relationship between CSR and financial performance [55], there has been no study estimating the impact of financial performance on CSR specifically in the Republic of Kazakhstan. Therefore, we hypothesize.

H1 Firm performance affects CSR practices in the Republic of Kazakhstan.

Exploring the hypothesis that firm performance affects CSR practices in the Republic of Kazakhstan is valuable for several reasons. Firstly, it allows us to understand this relationship within the unique context of Kazakhstan's rapid development, providing insights that can guide policymaking and business strategies. Secondly, it has practical implications for attracting foreign investment and shaping the future of CSR in the country. The country's rapid development and emergence in the global market make it an intriguing case study for understanding how CSR practices and financial performance are interrelated in emerging economies. This exploration is not only an academic endeavor but also a practical one, with the potential to shape the future of CSR in the Republic of Kazakhstan, contributing to its growth and sustainability.

The study aims to analyze the relationship between CSR activities and the performance of the largest companies in the Republic of Kazakhstan, which are included in the Index KASE list.

The contributions of this research can be summarised as follows.

1) Extends the investigation the correlation between CSR and firm performance indicators.
2) Helps to predict the development of CSR practices in the Republic of Kazakhstan.
3) The preliminary research investigates the relationship between firm performance and CSR practices in the emerging Kazakh market, following the post-Soviet era.

### 2.5 Conceptual framework

**Fig. 1:**
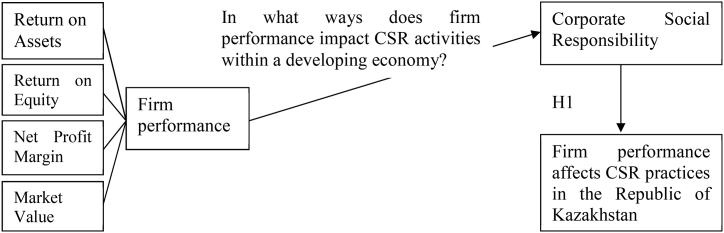
Conceptual framework diagram.

The conceptual framework of this research focuses on examining the intricate relationship between Corporate Social Responsibility (CSR) activities and firm performance within the Republic of Kazakhstan. The central research question driving this framework asks, "In what ways does firm performance impact CSR activities within a developing economy?" This framework is designed to offer a systematic approach for analyzing the bidirectional interactions between CSR initiatives and the performance of companies in Kazakhstan's distinctive socio-economic environment (see Fig. 1).

## 3 Materials and methods

The methodology employed in this study aims to establish a correlation between firm performance and Corporate Social Responsibility (CSR) activities in the Republic of Kazakhstan. The rationale behind our chosen methods is rooted in the objective of conducting this research in a developing country susceptible to external economic shocks. Emerging economies often face unique challenges in their business operations, and CSR activities are increasingly recognized to enhance corporate reputation and stakeholder engagement in such environments. For the purpose of this study, we utilized quarterly data from reliable sources, including Bloomberg (www.bloomberg.com) and the Kazakhstan Stock Exchange (www.kase.kz). This choice of data sources ensures the credibility and accuracy of the financial and market performance data we analyze. The study covers the performance of the eight largest corporations in the Republic of Kazakhstan over a ten-year period, spanning from Q1-2010 through Q1-2020.

The selection of Kazakh companies for inclusion in this study was based on several key criteria. These criteria included their track record of stable financial results, regular participation in stock exchanges, a commitment to corporate transparency, and the accessibility of public information for stakeholders. To ensure the representativeness of the sample and the diversity of economic sectors, companies from various fields of economic activity were included. The selected corporations encompassed a range of industries, such as Kcell (a mobile network operator), Halyk Bank (a financial and credit company), KazTransOil (an oil transit company), commercial bank BCC, Kaspi (an online services company), Kaztelecom (the national telecommunications company), KEGOC (an energy company), and Kazatomprom (the national atomic company). According to the KASE Index, these chosen companies collectively hold the largest market share in Kazakhstan, as illustrated in Table 1.

**Table 1:** Companies from index KASE^a^.

Company	Weight
Bank Center Credit	11.2 %
Halyk Bank	15.9 %
Kcell	16 %
KEGOC	9.7 %
Kaspi	15.7 %
Kazatomprom	14.1 %
Kazakhtelecom	11 %
Kaztransoil	6.4 %

a KASE Index is the ratio of included into the representative list shares market prices at the list development date to this list shares prices at a specific date, weighted on capitalisation considering free-floating shares.

In this study, CSR is quantified using Moskowitz's tripartite ratings, categorizing companies as 'outstanding,' 'honourable mentioned,' or 'worst' in terms of their CSR practices. This approach is based on Moskowitz's well-established categories, which have been utilized in previous studies by researchers like Cochran and Wood [34] and Sturdivant and Ginter [56]. The data pertaining to Moskowitz's categories for Kazakh companies were carefully hand-collected from annual audited reports covering the period from January 2010 to December 2020.

To analyze the data, we employed the least squares technique. The primary model is expressed as follows:

(1) CSR=α0+α1ROAt−1+α2ROEt−1+α3MVt−1+α4NPMt−1+ε

where.

*ROA* - Return on assets;

*ROE* - Return on equity;

*MV* - Market value;

*NPM* - Net profit margin.

In this model, we include previous financial performance as indicated by ROA (Return on Assets) and ROE (Return on Equity), which are traditional financial metrics used to measure profitability and operational efficiency. These indicators are critical in assessing a firm's financial health. We hypothesize that higher profitability and increased efficiency could positively affect CSR activities. This is based on the observation that more profitable companies tend to engage more actively in CSR initiatives, as supported by various studies [57,58]. This methodological approach was chosen to provide a comprehensive analysis of the relationship between CSR and firm performance while considering the specific economic context of Kazakhstan and its impact on corporate social responsibility practices.

## 4 Results

### 4.1 Descriptive statistics

Table 2 presents descriptive statistics parameters for all the variables under study in Kazakhstan. The parameters include mean, median, standard deviation, and interquartile range (IQR) for Return on Assets (ROA), Return on Equity (ROE), and Net Profit Margin (NPM).

**Table 2:** Descriptive Statistics (Kazakhstan's data).

Variable	Mean	Median	IQR
ROA	0.067	0.066	0.021
ROE	0.057	0.061	0.028
NPM	0.1025	0.1123	0.036

These parameters provide a comprehensive overview of the data spanning from Q1:2010 to Q4:2020. Additionally, incorporating the standard deviation offers a measure of how widely values vary from the average, helping to quantify the data's variability. The net profit margin, which is the ratio of net income to sales, and the return on assets, calculated as the ratio of net income to average total assets, are both key financial metrics included in our analysis. Total assets are logged using the natural logarithm to represent the asset variable. The analysis sets a significance level at 5 % to determine the statistical relevance of the findings.

### 4.2 Correlation analysis

Table 3 presents the correlation matrix for the variables under investigation. It includes correlation coefficients among Return on Assets (ROA), Return on Equity (ROE), Net Profit Margin (NPM), and Market Value (MV). The table reveals generally low correlations among the variables, with some relationships exhibiting negative values. This indicates an absence of significant multicollinearity problems, suggesting that all variables can be effectively analysed and utilized together in a single regression model.

**Table 3:** Correlation Matrix (Kazakhstan's data).

Variable	ROA	ROE	NPM	MV
ROA	1			
ROE	0.023	1		
NPM	0.012	0.005	1	
MV	0.010	0.012	0.043	1

The inclusion of significance levels and confidence intervals would enhance the interpretation of the correlations, providing information on the reliability and statistical significance of the relationships observed.

### 4.3 Unit root test

Before proceeding with the regression analysis, we conducted the Augmented Dickey-Fuller unit root test to identify any potential unit root issues. The test results revealed a unit root at the initial level. To address this issue, the data was transformed by logging and differencing in some cases, ensuring the stability and suitability of the dataset for further analysis.

### 4.4 Regression analysis

Table 4 illustrates the relationship between Corporate Social Responsibility (CSR) activities and profitability indicators in Kazakhstan. The accompanying T-test statistics in the table highlight the significance levels at both 5 % and 1 %, indicating the statistical relevance of the findings within these thresholds.

**Table 4:** CSR and firm performance indicators (Kazakhstan's data).

Significance level	5 %*	1 %**
ROA t-1	0.061	0.069
T-stat	1.821	1.674
P-value	0.000	0.000
ROE t-1	0.052	0.059
T-stat	2.932	2.741
P-value	0.000	0.000
NPM	0.451	0.049
T-stat	2.523	2.981
P-value	0.000	0.000
MV	0.002	0.002
T-stat	2.182	2.651
P-value	0.000	0.000
Scaled R squared	0.198	

These findings offer detailed insights into the relationships between financial and accounting ratios and CSR in Kazakh companies. Although Return on Assets (ROA), Return on Equity (ROE), and Market Value (MV) do not significantly influence CSR activities, there is a significant relationship between CSR and Net Profit Margin (NPM) in the Republic of Kazakhstan. To gain a more nuanced understanding of how CSR activities interact with financial performance, further analyses could involve subgroup examinations by industry sectors or company sizes. This approach would provide a deeper exploration of these dynamics within specific contexts.

## 5 Discussion

The main aim of this research was to analyze the relationship between Corporate Social Responsibility (CSR) and company performance in the Republic of Kazakhstan. Despite the considerable amount of research on the CSR-performance link, the findings across studies are generally inconsistent, as noted in sources [ [59,60]]. Our results are consistent with earlier findings [ [61]], which observed only modest success of CSR initiatives in the Kazakh corporate sector but noted a positive attitude towards companies' social contributions to all stakeholders. Our study concludes that in Kazakhstan, CSR extends beyond merely reacting to contemporary trends; it acts as a vital element in achieving competitive advantages, stabilizing social environments, and building trust among stakeholders and investors in Kazakh companies [ [62]].

The reveal that performance indicators such as Return on Assets (ROA), Return on Equity (ROE), and Market Value (MV) are not significantly correlated with CSR in the Republic of Kazakhstan. In contrast, Net Profit Margin (NPM) shows a strong association with CSR practices in the Republic of Kazakhstan. Although these findings may not align with prevailing scientific conclusions, they correspond to certain studies that have reported no significant linkage or even a negative correlation between CSR and firm performance [60]. Such patterns could be attributed to the unique characteristics of Kazakhstan's economic development. For instance, the conclusions drawn by Bhatia and Makkar [63] highlight the positive influence of CSR in developed countries, a viewpoint corroborated by by other research [ [15,27,28]].

The results indicate that Kazakhcompanies relate to CSR practices and firm performance, primarily through Net Profit Margin (NPM). Profitable companies are more actively involved in CSR practices in the Republic of Kazakhstan, and CSR practices are influenced by the performance of Kazakh firms. In this context, CSR practices in the Republic of Kazakhstan are more attractive to profitable businesses, those with higher NPM. These findings are noteworthy and distinct due to the Republic's status as an emerging post-Soviet economy.

Furthermore, we concur with the insights presented by Natsvlishvili [64], underscoring that the CSR phenomenon is relatively new for post-Soviet countries. In these nations, the state traditionally bore the responsibility for the Environmental, Social, and Governance (ESG) triad and managed it in a systematic manner. New companies are investing in CSR projects, but often in a haphazard fashion, leading to reduced CSR effectiveness.

## 6 Implications

### 6.1 Theoretical implications

This study significantly contributes to the theoretical understanding of the relationship between Corporate Social Responsibility (CSR) and firm performance. Our findings, identifying a positive correlation in the Republic of Kazakhstan, are in line with established CSR theories, offering empirical evidence that CSR practices can enhance financial performance. This alignment reinforces the notion that socially responsible actions can create substantial value for companies operating in emerging economies. Moreover, our results suggest that theoretical frameworks within CSR should be adapted to accommodate the specific contexts of emerging markets. This adaptation involves recognizing and incorporating unique factors such as regulatory environments and stakeholder expectations. By contextualizing CSR theories within the dynamics of emerging economies like Kazakhstan, scholars can develop more nuanced and applicable frameworks that accurately capture the complexities of CSR implementation and its impact on firm performance in these settings.

### 6.2 Practical implications

The positive correlation between CSR practices and firm performance implies that investing in CSR initiatives can be strategically beneficial for companies and enhance financial performance and competitiveness. This may involve targeted initiatives focusing on sustainability, employee well-being, and community engagement. Such efforts not only contribute to societal well-being but also elevate brand image and reputation, leading to improved financial outcomes. These findings have practical implications for industry practices, suggesting that companies can gain a competitive edge by prioritizing CSR, especially in emerging markets like Kazakhstan. Demonstrating commitment to social responsibility can attract socially conscious consumers and build long-term stakeholder relationships. Policymakers can incentivize CSR through supportive policies, tax incentives, and transparent reporting requirements. Furthermore, our research highlights the need for further exploration of how CSR influences firm performance in emerging market contexts.

## 7 Conclusions

The findings of this study affirm a positive correlation between CSR practices and firm performance, aligning with established theoretical perspectives. In summary, this research underscores the importance of CSR activities in emerging economies, particularly in the Republic of Kazakhstan. The demonstrated positive correlation between CSR practices and financial performance underscores the potential advantages for companies operating within these regions. It is imperative for both the academic community and business practitioners to consider the implications of these findings, paying special attention to the distinctive characteristics of emerging markets and the long-term effects of CSR initiatives on financial performance. As the global business environment continues to evolve, the integration of CSR into business strategies is increasingly crucial for enduring success and societal progress.

## 8 Limitations and directions for future research

It is crucial to recognize the limitations of this study. Firstly, the research was based on a relatively small sample size, encompassing only eight firms. A larger sample would offer a more extensive insight into the relationship between CSR and firm performance in the Republic of Kazakhstan. Moreover, the study spanned a limited timeframe of ten years, potentially not capturing the long-term impacts adequately. Another challenge was data availability, as certain data points were inaccessible within the Republic of Kazakhstan.

This study paves the way for future research in the field of CSR and firm performance. Building on these initial findings, future research could examine the relationship between CSR and market-based performance metrics, such as stock prices and market indexes. Further studies might utilize advanced analytical techniques like the Granger causality method to explore the reciprocal influence between CSR and profitability. It would also be beneficial to incorporate additional variables, such as Retained Earnings, in subsequent research. Comparative analyses across developing and developed countries could provide deeper insights into the global relevance of these findings. Additionally, employing qualitative research methods like in-depth interviews and surveys could enrich the quantitative data, offering a more nuanced understanding of the intricate relationship between CSR and financial performance.

## Ethics approval and consent to participate

Not applicable.

## Data availability statement

No data associated with our study has been deposited into a publicly available repository. For primary data sources, information will be provided on request.

## Funding

This research received no specific grant from funding agencies in the public, commercial, or not-for-profit sectors.

## CRediT authorship contribution statement

**Maya Katenova:** Writing – original draft, Investigation, Formal analysis, Data curation. **Hassan Qudrat-Ullah:** Supervision, Resources, Project administration, Methodology, Investigation, Conceptualization.

## Declaration of competing interest

The authors declare that they have no known competing financial interests or personal relationships that could have appeared to influence the work reported in this paper.
